# Navigating pregnancy, postpartum care and reproductive autonomy with sickle cell disease in Uganda: an interpretive phenomenological study in Eastern and Central Uganda

**DOI:** 10.21203/rs.3.rs-9692986/v1

**Published:** 2026-06-02

**Authors:** Jackline Akello, Grace Ndeezi, Annette Nakimuli, Musa Sekikubo, Ian Munabi, Ruth Namazzi, Deogratias Munube, David Mukunya, Pebalo Francis, Kenneth Mugabe, Savio Mwaka, Joseph Rujumba, Sarah Kiguli

**Affiliations:** Makerere University; Makerere University; Makerere University; Makerere University; Makerere University; Makerere University; Makerere University; Busitema University; Gulu University; Mbale Regional Referral Hospital; Uganda Institute of Information and Communication Technology; Makerere University; Makerere University

**Keywords:** sickle cell disease, pregnancy, postpartum care, reproductive autonomy, maternal health, qualitative research, Uganda

## Abstract

**Introduction:**

Pregnancy and the postpartum period are high-risk transitions for women living with sickle cell disease (SCD), yet evidence from Uganda has focused largely on clinical outcomes rather than women’s lived experiences of reproductive decision-making and maternity care. This study explored how pregnant and postpartum women with SCD in Central and Eastern Uganda experienced pregnancy, postpartum care, reproductive counselling, social support, stigma and coping.

**Methods:**

We conducted an interpretive phenomenological qualitative study at Kawempe National Referral Hospital and Mbale Regional Referral Hospital. Thirty-five unique women with confirmed SCD participated: 25 in in-depth interviews and 10 additional women in two site-based focus group discussions. Participants were purposively selected to capture variation by pregnancy status, postpartum status, parity, education, marital status, site and pregnancy outcome. Interviews and discussions were audio-recorded, transcribed verbatim, translated into English where necessary, de-identified and analyzed thematically using an interpretive phenomenological approach informed by a socio-ecological lens.

**Results:**

Women described pregnancy with SCD as a complex reproductive and health-system experience shaped by fear, family responses, financial vulnerability, provider attitudes, fragmented referral pathways and wider social norms. Five interrelated themes were identified: navigating fragmented and poorly coordinated care; financial strain and inconsistent access to medicines, blood and investigations; provider attitudes, limited communication and reproductive counselling; emotional distress, uncertainty and fear for self and baby; and coping through family support, peer networks, faith and selfmanagement. Myths that people with SCD do not survive into adulthood or should not reproduce contributed to secrecy, stigma and delayed care-seeking. Participants recommended multidisciplinary SCD-responsive antenatal and postpartum services, clear referral pathways, respectful counselling, reliable supplies, financial protection and strengthened psychosocial support.

**Conclusion:**

Women with SCD require maternity care that goes beyond risk avoidance. Integrated, respectful and multidisciplinary services that combine clinical preparedness with reproductive autonomy, psychosocial support and continuity across pregnancy, delivery and postpartum care could improve women’s experiences and outcomes in Uganda.

## Background

Sickle cell disease (SCD) is a hereditary blood disorder marked by chronic anaemia, vaso-occlusive crises, and complications affecting multiple organs. It disproportionately impacts people of African, Mediterranean, Middle Eastern, and Indian descent (1). Medical advancements have enhanced life expectancy for those with SCD, resulting in more women reaching reproductive age and seeking maternal healthcare. However, pregnancy in women with SCD is still considered high-risk, accompanied by greater maternal and perinatal morbidity and mortality due to complications such as preeclampsia, preterm labour, intrauterine growth restriction, and damage to maternal organs (2, 3). Despite these dangers, there is limited research on the experiences of pregnant and postpartum women with SCD, especially in low-resource areas where healthcare access may be challenging. Accessing maternal healthcare is incredibly challenging for women with SCD, who often face complicated healthcare systems filled with systemic obstacles, including geographical barriers, financial challenges, and fragmented care(4, 5). Provider biases, stigma, and misunderstandings about the reproductive abilities of women with SCD further deepen healthcare disparities(6). The psychological stress of pregnancy, which involves increased anxiety and fear of adverse maternal and neonatal outcomes, complicates their situations(7).

Existing qualitative evidence is limited but useful for positioning this study. A Ugandan phenomenological study at Mulago National Referral Hospital found that women with SCD experienced both individual and health-system challenges during pregnancy, including pain crises, pregnancy loss, stigma, discouragement, relationship strain, delays in receiving care, informal costs and variable support from health workers(8). A Brazilian exploratory study similarly showed that pregnancy among women with sickle cell anaemia was shaped by ambivalence, fear, social judgement and concerns about maternal and fetal risk(9). An integrative review of reproductive decision-making in women with SCD also highlighted how decisions about pregnancy are influenced by genetic risk, partner and family relationships, prior health-care experiences, and the availability of credible counselling(10). More recent qualitative work on contraception among women with SCD shows that reproductive counselling remains uneven and that women require balanced, autonomy-supporting information rather than directive messages to avoid pregnancy(11). These studies demonstrate the importance of lived experience, but they are limited by single-site designs, limited exploration of postpartum experiences, and insufficient attention to how individual, interpersonal and health-system factors interact across referral settings. In Uganda, there remains paucity of multisite qualitative evidence on these experiences and coping strategies among pregnant and postpartum women with SCD. This study addresses that gap by examining experiences across two referral hospitals in Central and Eastern Uganda.

This study explored the lived experiences of pregnant and postpartum women with SCD in Uganda, with particular attention to reproductive decision-making, maternity-care navigation, social support, stigma and coping during pregnancy and the immediate postpartum period.

## Methods

This was a qualitative study informed by interpretive phenomenology, which was appropriate for exploring how women made meaning of pregnancy, childbirth and postpartum recovery while living with SCD. The phenomenon of interest was the lived experience of navigating pregnancy and postpartum care with SCD, including care-seeking, communication with providers, reproductive decision-making, stigma, social support and coping. The sample size was guided by information power and saturation, recognizing that sample adequacy in qualitative research depends on the specificity of the study aim, the specificity of participants, the quality of dialogue and the depth of analysis rather than numerical representativeness alone(12, 13). Data were generated from 35 unique women: 25 participated in indepth interviews (IDIs), and 10 additional women participated in two site-based FGDs. Semi-structured IDI and FGD guides were used to explore both personal and shared experiences. Reporting was reviewed against the Consolidated Criteria for Reporting Qualitative Research (COREQ) to strengthen transparency in qualitative reporting(14).

### Study setting and data collection context

The study was conducted at Kawempe National Referral Hospital in Central Uganda and Mbale Regional Referral Hospital in Eastern Uganda between December 2024 and May 2025. Both hospitals are teaching and referral facilities that provide comprehensive maternal and neonatal care services and receive women with high-risk pregnancies, including critically ill obstetric patients and women living with SCD. Participants were recruited from the high-risk antenatal clinic, postnatal ward and gynaecology ward, including women who had experienced pregnancy loss.

Interviews were conducted in the participant’s preferred language. The semi-structured interview and focus group discussion guides were developed specifically for this study and have not been published elsewhere; the English-language version is provided as Supplementary File 1. At Kawempe National Referral Hospital, interviews were conducted in English or Luganda, while at Mbale Regional Referral Hospital they were conducted in English or Lumasaba. IDIs typically lasted 45–60 minutes, while FGDs lasted approximately 60–90 minutes. This duration allowed sufficient time to explore individual experiences in depth during interviews and to capture shared meanings, group interaction and collective recommendations during FGDs.

### Study Population

The study population comprised pregnant and postpartum women with confirmed SCD who sought care at the two study sites during the study period. SCD diagnosis was based on prior documented haemoglobin electrophoresis results or clinical history suggestive of SCD followed by haemoglobin electrophoresis conducted through the hub system. To capture variation in experience, the study included women with different pregnancy outcomes, including live births, stillbirths, abortions, vaginal deliveries and caesarean sections. It also included women of different gravidity and parity, ranging from primigravidae to multigravidae, to enrich the accounts of first-time motherhood, repeated pregnancy, and pregnancy after prior adverse outcomes.

### Sampling

The study used purposive sampling with a maximum-variation strategy to include women with diverse social and clinical experiences. Variation was sought by marital status, parity, socioeconomic status, education level, pregnancy status, postpartum status, place of care, and pregnancy outcome. Pregnant and postpartum women with SCD were identified through the maternity and sickle cell service points at the two hospitals and invited to participate in either IDIs or FGDs depending on their availability, preference, clinical stability and suitability for private or group discussion.

### Procedure for data collection

Data collection was guided by information power and saturation. IDIs were used to elicit detailed personal accounts of pregnancy, delivery, postpartum recovery, care-seeking and sensitive reproductive experiences. FGDs were used to explore shared meanings, community-level perceptions, myths, stigma and collective recommendations for improving care. To avoid inflating the apparent sample size, each participant was counted once in the overall total. The final sample comprised 25 IDI participants and 10 FGD participants, giving 35 unique participants across the two study sites. The two FGDs were conducted as site-based group discussions, one at Kawempe National Referral Hospital and one at Mbale Regional Referral Hospital, with five participants per group. This structure allowed the study to retain individual depth through IDIs while also capturing interaction and shared meaning through FGDs. Interviews and FGDs were conducted by the principal investigator and a trained research coordinator in private rooms within the outpatient or maternity service areas where participants received care. With permission, all sessions were audio-recorded, transcribed verbatim, translated into English where necessary, and de-identified. Field notes and reflexive journals were maintained to document contextual observations, interviewer reflections, emerging interpretations and potential biases. Recruitment continued until no substantially new themes were emerging across methods and study sites.

### Data analysis

Transcripts were analysed using thematic analysis informed by interpretive phenomenology. The analysis combined inductive coding of participants’ accounts with sensitising concepts from the socioecological framework to distinguish individual, interpersonal, health-system and wider societal influences. At least two members of the research team independently read a subset of transcripts, developed an initial codebook and refined it through discussion. Coding differences were resolved through consensus, and emerging categories were compared across IDIs, FGDs, pregnancy status, postpartum status and study site. Reflexive notes were used to examine researcher assumptions and strengthen interpretation. Credibility was enhanced through team debriefing, use of verbatim quotations, triangulation across IDIs and FGDs, and maintaining an audit trail of coding decisions.

## Results

### Participant Demographics

A total of 35 unique women participated in the study across the two sites: 25 women participated in IDIs and 10 additional women participated in two FGDs. The IDIs were distributed across the two referral settings to capture individual experiences from both Central and Eastern Uganda. The two FGDs were conducted as site-based discussions, one at Kawempe National Referral Hospital and one at Mbale Regional Referral Hospital, with five participants in each group. Complete structured characteristic data were available for the 25 IDI participants and are summarised in Table 1. Among these 25 participants, 15 were pregnant and 10 were postpartum or post-abortion. Participants were aged 19–34 years, with a mean age of 24 years. Educational attainment varied: 12 (48%) had attained primary-level education, 9 (36%) had attained secondary-level education and 4 (16%) had attained tertiary-level education or higher. Nine participants (36%) were unmarried and living with relatives or parents, while 16 (64%) were married or cohabiting and living with partners. Table 1 also summarises the qualitative data-collection profile by method and study site. Quotations are labelled by method, participant number and site to show distribution across data sources while preserving confidentiality.

The major themes and sub-themes are presented in Table 2 and organised using a socio-ecological lens. This structure distinguishes individual-level experiences, interpersonal and community influences, health-system barriers, wider societal norms, and participant recommendations for improving care.

#### Navigating the healthcare system

##### Access to Care

At the health-system level, women described care-seeking as a process of moving between facilities, clinics and providers while trying to secure timely, specialised and respectful maternity care. The accounts below therefore combine practical barriers to access with women’s perceptions of whether the system recognised SCD in pregnancy as requiring urgent and coordinated attention.

Participants reported a wide range of experiences regarding access to healthcare services. Many women described difficulties securing timely review by clinicians familiar with SCD in pregnancy. One participant articulated a common experience: “*It was hard to get seen by a doctor who understood my condition. I was referred from Kawala Health Centre IV to Kawempe Hospital. Then Kawempe Hospital also referred me to Mulago Specialised Women’s Hospital. I felt like everywhere I went; they just wanted to refer me to another level. It was so frustrating that I had to wait weeks. By then, I was already in crisis*” (IDI-19, Kawempe).

Other barriers included long waiting times in antenatal clinics, lack of triage for women presenting with pain or acute symptoms, absence of dedicated antenatal services for women with SCD, and negative provider attitudes. Several participants felt that their pain was dismissed or misunderstood, which delayed appropriate care and contributed to mistrust. One postpartum mother reflected, “*I felt like I had to prove my pain all the time in order to gain attention. Sometimes I did not even want to go to the hospital because I knew they would think I was just being dramatic*” (IDI-07, Mbale).

##### Financial Constraints

The financial burden of managing sickle cell disease during pregnancy was a prominent theme in the discussions. Participants reported significant out-of-pocket expenses related to medications, regular check-ups, and emergency interventions including paying for caesarean sections and ultrasound scans. Many women highlighted the high costs of medication necessary for managing their condition, particularly the cost of clexane, a low molecular weight heparin which is part of their treatment when they are admitted, which often places a strain on their financial resources. One participant shared, “*In Kawempe the doctors see you very well, but you have to buy most of the medicines you need, and then there is that drug Clexane, which is expensive*” (FGD-Kawempe-P4).

The necessity of purchasing medications, coupled with the limited availability of subsidised options, led to difficult choices about healthcare access. Some women reported forgoing medicines or skipping review appointments because of cost. In addition to medication costs, participants discussed the financial implications of transport to referral facilities. One mother noted, “*Every time I had to go for ANC, it cost me a lot just to get there. Sometimes I had to skip appointments because I could not afford transport, or I would forego meals just to transport myself to Kawempe, which also affected my health*” (IDI04, Kawempe). These financial burdens affected health-seeking behaviour and contributed to frustration and helplessness.

Additionally, access to blood transfusions emerged as a critical issue for participants, particularly for those who experienced severe complications during pregnancy. The availability of compatible blood was reported as inconsistent, which raised concerns about the potential risks associated with delayed transfusions. Participants shared experiences of being told that blood was unavailable when they required urgent transfusions. The fear of not receiving timely care due to blood shortages was a significant concern that underscored the need for improved blood bank resources and management.

##### Lack of communication and coordination

Continuity of care emerged as a significant concern. Participants reported limited communication and coordination between providers involved in their care, particularly between antenatal, haematology, emergency, delivery and neonatal services. This fragmentation resulted in inconsistent treatment plans, repeated referrals and a sense of being “lost” within the health system. Several women wanted an integrated approach in which obstetricians, haematologists, anaesthetists, neonatologists and nurses developed a shared care plan. As one mother stated, “*I was referred to Mulago Women’s Hospital to be delivered and my baby was taken to the nursery, but when I reached, they changed my treatment plan. I was just confused*” (IDI-08, Kawempe).

##### Provider Attitudes

Participants’ experiences with healthcare providers varied. Some women reported positive interactions with empathetic and knowledgeable providers who listened to their concerns. One participant stated, “*My doctor was amazing. She really listened and made me feel like my health mattered*” (FGD-Mbale-P2). Conversely, negative experiences were also common. Some participants described perceived bias or discrimination, particularly during crowded antenatal clinic visits where providers were seen as rushed, dismissive or rude.

Many women reported feeling pressured by healthcare professionals to avoid pregnancy because of perceived risks. One participant stated, “*I was told that I was not supposed to get pregnant. It made me feel like I had no choice*” (FGD-Mbale-P5). This discouragement created helplessness and anxiety about reproductive choices. Despite the discouragement, participants wanted family planning options and counselling that would enable informed decision-making. Many wished to receive balanced information on the risks, benefits and practical preparation required for pregnancy with SCD, rather than being advised only not to conceive.

##### Communication and education

Many participants expressed a desire for more educational resources, particularly regarding selfmanagement, pain management, labour, delivery and postpartum recovery. One participant stated, “*I would have liked more information on what to expect during labour and delivery. It felt like I was going in blind. The health workers have no time to tell us much. The health education sessions in ANC are for general high-risk conditions, nothing specific to us with sickle cell disease*” (FGD-Kawempe-P1). The lack of SCD-specific educational materials was particularly concerning for participants because general antenatal health talks rarely addressed pain crises, hydration, danger signs, delivery preparation, newborn care, contraception or postpartum recovery for women living with SCD.

#### Emotional and Psychological Impact

##### Anxiety, Uncertainty and Fear for self and baby

The emotional toll of navigating pregnancy with SCD was a recurring individual-level theme. Participants described anxiety, uncertainty and fear about their own health, the survival of their babies, pain crises, blood shortages, delivery complications and whether they would be able to care for a newborn while living with SCD. One participant articulated, “*Every time I felt a pain, I worried it was something serious. The fear of complications was always there” (IDI-06, Mbale). A postpartum mother remarked, “I wish someone had sat down with me and explained everything. I felt like I was just left to learn from others through rumours*” (FGD-Kawempe-P4).

The lack of clear information from healthcare providers compounded this anxiety. Many participants reported feeling uninformed about the potential risks associated with sickle cell disease during pregnancy, leading to increased stress. Fear emerged as a critical factor influencing mental health and overall well-being, affecting both the pregnancy experiences and postpartum adjustments. The fear of stigmatization and misunderstanding from healthcare providers, community, and family members emerged as a significant concern. Many expressed apprehensions about disclosing the pregnancy, fearing that they would be viewed as incapable or overly fragile. A participant noted, “*I did not want people to know I was pregnant. I was scared they would not understand my struggles, especially since I was working as a house help to survive*” (IDI-03, Mbale).

This fear of stigma affected participants’ interactions and their willingness to seek support and care. In several accounts, stigma was anticipated rather than directly experienced, while other narratives described enacted stigma through family rejection, discouragement from pregnancy, dismissal of pain, or being treated as incapable. Participants also feared complications affecting their unborn babies, inability to care for newborns, and failure to meet social expectations of motherhood. These accounts show how biological risk, motherhood, poverty and social judgement intersected to shape women’s mental health, underscoring the need for psychological support, respectful care and clear patient education.

##### Coping Strategies and Resilience

Participants demonstrated a range of coping strategies to manage the challenges associated with sickle cell disease during pregnancy and postpartum.

At the interpersonal level, support from partners, parents, siblings, peers and other women with SCD shaped how participants coped with pregnancy and postpartum recovery. Support was practical, emotional and financial, but its absence could intensify isolation and fear.

The importance of support systems emerged as a critical factor in managing the emotional challenges of pregnancy and postpartum recovery. Participants highlighted the role of family, friends and support groups in providing emotional and practical support. One participant noted, “*Having someone who understood what I was going through made all the difference. My sister was always there for me; even when I delivered, she was the one who took care of the baby*” (FGD-Kawempe-P1).

However, not all participants had access to such support. Some reported feelings of isolation, particularly in the postpartum period, when the physical and emotional demands of caring for a newborn compounded their health challenges. One mother shared, “*I felt so alone after giving birth. It was hard to find other mothers who understood what I was going through with sickle cell. Even my family looked tired of looking after me and the baby. I would just cry alone in silence”* (IDI-10, Mbale).

##### Community Engagement

Community engagement emerged as a vital aspect of resilience for participants. Many women spoke about the positive impact of connecting with others who shared similar experiences through formal support groups or informal networks. One participant stated, “*Being part of a group where everyone understands what you are going through is incredibly powerful. It makes you feel less alone. I have been part of the Mulago sickle cell clinic and I feel more welcomed there than here in Kawempe*” (FGD-Kawempe-P2). Participants also expressed a desire for more community-based resources tailored to the needs of pregnant women with SCD. The importance of peer support was underscored, with many women advocating for mentorship programmes that connect expectant mothers with those who have successfully navigated similar challenges.

##### Myths and Misconceptions

The FGDs and IDIs revealed that myths and misconceptions about SCD significantly affected the lived experiences of pregnant and postpartum women. These misconceptions shaped social interactions, mental health, reproductive choices and perceptions of healthcare, and operated at family, community and provider levels.

Participants reported societal misconceptions surrounding SCD, which often led to stigma and discrimination. Many women described being treated differently by families, communities and sometimes providers because of their condition. A common myth was that people with SCD cannot survive beyond a certain age, so resources, particularly education, should not be invested in them. One participant stated, “*My parents told me that I would not live beyond 19 years, so they could not waste money educating me. I never went to school while my siblings without SCD were educated, and now I am here, 32 years, uneducated and jobless*” (IDI-03, Mbale).

A significant misconception highlighted by participants was the belief that women with SCD should not become pregnant. Several women shared that they faced pressure from family members and friends who advised against pregnancy due to fears about complications. One participant stated, “*My mother was angry when I told her I was pregnant. She thought I would die in childbirth. She abandoned me with my grandmother and up to now I have not seen her*” (IDI-06, Mbale).

Participants also reported receiving conflicting information from healthcare providers, which contributed to anxiety. “One doctor told me that I should not have children, but another said it was possible with the proper care. It was very confusing, so I opted to continue getting care from Mulago Children’s SCD clinic because they were more organised, despite the pregnancy” (IDI-07, Kawempe). This ambiguity contributed to helplessness and uncertainty regarding reproductive choices.

##### Perceived Impact of SCD on Maternal and Infant Health

This theme reflected women’s interpretation of risk, not only biomedical risk. Participants often understood SCD and pregnancy through stories of maternal death, infant loss, emergency transfusion, severe pain, or inability to care for a baby after birth.

The participants also discussed their beliefs about the impact of SCD on their health and that of their infants. Many women feared that their condition would lead to poor outcomes for their babies and themselves, as most examples they were given were of women who had died or lost their babies. Moreover, participants often mentioned that healthcare providers focused on the risks associated with SCD rather than providing comprehensive care and support. They felt like the healthcare providers worried more about the complications of SCD rather than caring for them as a whole human being.

##### Recommendations for Healthcare Improvements

Based on their experiences, participants provided several recommendations aimed at improving the healthcare system for pregnant and postpartum mothers with sickle cell disease.

Participants recommended financial protection for women with SCD, including subsidies for essential medicines, investigations, transport and emergency obstetric care. They suggested that hospitals and partner organisations consider mechanisms to support women who cannot afford care. They also emphasised reliable availability and timely administration of essential medicines, including pain medicines and low-molecular-weight heparin when clinically indicated, because many women present in acute states and may be unable to purchase medicines immediately. Participants further recommended dedicated or clearly linked antenatal services for women with SCD, with multidisciplinary input from obstetrics, haematology, anaesthesia, neonatology, nursing, counselling and social work. Such services could provide tailored health education, individual care plans, triage, regular monitoring, clear referral pathways and continuity across pregnancy, delivery and postpartum care. The need for hydration support, including access to safe drinking water on wards, was also emphasised because dehydration was perceived to worsen symptoms during labour and postpartum recovery. Finally, participants called for comprehensive reproductive health counselling that respects autonomy while providing clear, individualised information about pregnancy risks, contraception, birth spacing and preparation for future pregnancies.

## Discussion

This qualitative study illuminates the multifaceted lived experiences of pregnant and postpartum women with SCD in Eastern and Central Uganda. The findings show that pregnancy with SCD was experienced not only as a clinical high-risk condition but also as a social, emotional and health-system navigation challenge. Using a socio-ecological interpretation, women’s experiences were shaped by individual fears and coping strategies, family and community responses, health-system barriers, and broader myths and norms about SCD, survival and reproduction.

One predominant theme identified was the challenges participants faced in navigating the healthcare system. Many women reported difficulties accessing appropriate care, often worsened by a lack of awareness and understanding of SCD among healthcare providers. This finding aligns with existing literature highlighting systemic barriers to healthcare access for individuals with chronic conditions(15, 16). Access to healthcare is a critical determinant of health outcomes for those with SCD; thus, geographic limitations, socioeconomic disparities, and a lack of specialised services not only delay appropriate care but also exacerbate patients’ conditions. Financial barriers significantly impact the ability of SCD patients to seek and maintain care(16). The high costs associated with treatments, medications, and hospitalizations can discourage patients from accessing necessary services (17). Many patients reported that the financial burden influenced their decision to delay or forgo treatment, sometimes leading to poor health outcomes. Health insurance coverage plays a crucial role in mitigating these financial constraints; however, disparities in coverage can exacerbate inequalities in access to care (17).

Many participants in our study expressed frustration with repeatedly explaining their condition to various providers, which reflects the fragmented, inconsistent, and poor continuity of care. This finding is also emphasized by who noted that uncoordinated care results in suboptimal treatment outcomes(18). Ineffective communication among providers can lead to fragmented care, causing mismanagement of the disease and increased hospitalizations. Establishing clear communication channels and care pathways improves continuity of care and ensures that patients receive thorough and cohesive treatment.

Furthermore, provider attitudes toward patients with SCD can significantly impact patient experiences within the healthcare system. Similar findings have been reported in the literature, where individuals with SCD are often seen as drug-seekers, and their pain crises are dismissed(6). Research shows that some healthcare providers hold misconceptions about the disease, leading to stigmatization and insufficient treatment(17). These negative attitudes can cause patients to feel marginalized and reluctant to seek care, further intensifying health disparities Despite negative provider attitudes, some women have reported positive experiences with empathetic and knowledgeable healthcare providers. When clinicians adopt a patient-centred approach and encourage open communication, patients with chronic illnesses feel more supported. Interventions, such as provider education and sensitivity training, have proven effective in reducing bias and enhancing patient-provider relationships(16). Training programmes to increase provider knowledge and empathy regarding SCD are crucial for creating a more supportive healthcare environment.

The theme of emotional and psychological issues was also prominent in the findings. Participants frequently described feelings of anxiety, fear, and isolation, stemming not only from the physical challenges of SCD but also from the societal stigma associated with the disease. The unpredictable nature of SCD can lead to significant emotional distress for both patients and their families. The uncertainty about disease progression, pain episodes, and potential complications can contribute to increased anxiety. Studies have shown that patients with SCD often experience elevated rates of anxiety and depression, which can complicate disease management further(7). Fear of pain crises, hospitalizations, and the potential for long-term complications can significantly affect the quality of life for individuals with SCD. This fear often leads to avoidance behaviours, such as reluctance to engage in physical activities or social interactions, ultimately isolating patients and worsening their emotional distress(7, 19). The psychological burden of managing a chronic illness during pregnancy can be profound, impacting both maternal mental health and the overall pregnancy experiences(20). Mental health support services, including counselling and support groups, can provide a valuable outlet for patients to address their fears and develop coping strategies. Healthcare providers must recognise these emotional dimensions and incorporate mental health support into the care regimen for pregnant women with SCD. Developing multidisciplinary care models that include mental health professionals could offer a more holistic approach to treatment, addressing both the physical and psychological needs of these women(20).

Participants discussed various coping strategies they used to manage their condition and handle the complexities of pregnancy. These strategies included seeking support from family and friends and practicing self-care. Notably, many women highlighted the importance of community support, which is critical in building resilience. This aligns with broader research on coping mechanisms among those with chronic illnesses, indicating that community involvement can markedly improve quality of life for these women(16). Studies show that resilience can be significantly enhanced through personal coping strategies for patients with SCD Support systems consisting of families, friends, and community connections are essential in helping individuals with SCD face the emotional and physical hurdles of the disease. Strong social networks offer emotional support, practical help, and a sense of belonging, vital for overall well-being(21, 22). However, in specific cultural settings, stigma associated with SCD can lead to social isolation instead of support(6). Community-centred programmes that foster social ties and engagement can strengthen these support systems. Future initiatives should aim to use community resources to build networks that empower women living with SCD. Many participants expressed feeling dissuaded from pregnancy by their healthcare providers. providers due to perceived risks of SCD in pregnancy. This discouragement leads to feelings of shame and inadequacy, impacting patients' overall emotional well-being and their reproductive choices. Literature supports this finding, with Research indicates that providers often advise against pregnancy rather than offering personalized risk assessments(23). Tumwesige and colleagues explored the lived experiences of pregnant women with sickle cell disease at Mulago Hospital. They revealed challenges such as stigma, discouragement, informal costs, and abandonment by partners(8). Although many providers dissuade pregnancy for SCD patients, participants pointed out that limited contraceptive counselling and access to safe methods is a significant issue. Misinformation regarding hormonal contraceptives and their effects on SCD remains persistent among both patients and healthcare providers(24). Access to contraceptive services and comprehensive reproductive health information is crucial for those with SCD. Unfortunately, many patients encounter challenges in finding reliable information on contraceptive options and family planning(24). This informational gap can lead to unintended pregnancies and associated complications, emphasizing the need for enhanced reproductive health education and resources specifically designed for individuals with SCD.

Cultural myths, such as the view that women with SCD should avoid having children, contribute to stigma and frequently impact reproductive choices. Additionally, misinformation regarding genetic transmission and pregnancy risks was widespread among participants. Similar issues have been highlighted in studies indicating that misunderstandings about SCD inheritance and maternal mortality lead to discrimination (23). Healthcare providers must confront these misconceptions through education and open dialogue to empower patients to make informed choices regarding their reproductive health(17).

Moreover, participants suggested various ways to enhance the healthcare experience for pregnant women with SCD. Their suggestions included improving educational resources, increasing accessibility to healthcare services, and creating support groups specifically designed for pregnant women with SCD. These recommendations emphasize the significance of including patients in formulating healthcare policies and programmes, ensuring that their voices and needs are acknowledged.

## Conclusion

This study shows that pregnancy and postpartum recovery among women with SCD in Uganda are shaped by clinical risk, fragmented referral pathways, financial barriers, stigma, family responses and limited reproductive counselling. Women’s accounts demonstrate that counselling should not be limited to discouraging pregnancy or emphasising risk. Instead, maternity care should combine honest discussion of risk with reproductive autonomy, birth preparedness, pain and crisis management, psychosocial support, partner and family engagement, and continuity of care across antenatal, delivery and postpartum services. Establishing multidisciplinary SCD-responsive maternity pathways at referral hospitals, supported by reliable supplies of essential medicines and blood products, could improve both women’s experiences and maternal and newborn outcomes.

## Figures and Tables

**Table 1:** Participant profile, data-collection methods and available structured characteristics

Parameter	IDIs	FGDs	Overall note
Number of sessions	25	2	27 qualitative sessions.
Unique participants	25 women	10 additional women	35unique participants; no double counting.
Study sites	Kawempe and Mbale	One FGD per site; five participants per FGD	Two referral contexts: Central and Eastern Uganda.
Purpose	Individual depth on pregnancy, care-seeking, delivery, postpartum recovery and coping	Shared meanings on myths, stigma, social support and care recommendations	Methods were complementary: IDIs gave depth; FGDs captured interaction and shared meaning.
Eligibility	Pregnant or postpartum/post-abortion women with confirmed SCD	Pregnant or postpartum/post-abortion women with confirmed SCD	Confirmation was by documented electrophoresis
Sampling	Maximum variation by marital status, parity, education, socioeconomic status, pregnancy status, postpartum status, site and outcome	Maximum variation by shared care experience, stigma, support systems and recommendations	Purposive sampling enriched the range of accounts.
Structured characteristics	Detailed demographic and clinical characteristics available for the 25 IDI participants.	FGD participants were summarized by site and method; individual clinical characteristics were not used as a quantitative denominator.	Table denominators are stated explicitly to avoid double counting across methods.
Quotation identifiers	IDI identifiers include method, participant number and site, for example IDI-04, Kawempe.	FGD identifiers include method, site and participant number, for example FGD-Mbale-P3.	Identifiers show both method and site while preserving participant confidentiality.

**Table 2:** Themes and subthemes derived

Level	Theme	Subthemes
Health system	Barriers to accessing and sustaining care	Long waiting times and lack of triage in ANC clinics
		Out-of-pocket costs and financial constraints
		Blood shortages
		Fragmented referral systems, poor coordination, and loss of clinical information
		Provider attitudes and interpersonal care
		Inadequate communication and patient education
		Provider discouragement regarding pregnancy versus limited contraceptive counselling
Individual	Emotional and psychological burden	Anxiety and uncertainty
		Fear related to pregnancy outcomes and disease complications
	Coping strategies and resilience	Family and social support systems
		Self-management and adaptive behaviours
		Engagement in community-based support activities
Societal	Myths, stigma, and social norms	Belief that individuals with SCD do not survive into adulthood
		Norms discouraging reproduction among women with SCD
		Stigma limiting educational opportunities for individuals with SCD
Health system recommendations	Suggested strategies for improving care	Financial protection mechanisms for patients
		Strengthening blood donation systems
		Subsidizing costs of medicines and services
		Comprehensive reproductive health counselling
		Establishment of specialised ANC services for women with SCD

## Data Availability

https://doi.org/10.7910/DVN/F3BVEZ De-identified excerpts supporting the findings are included in this article. Full qualitative transcripts are not publicly available because they may contain potentially identifiable contextual information. Additional de-identified data may be made available from the corresponding author on reasonable request and with relevant ethical permissions.

## Abbreviations

ANC: Antenatal Care
FGD: Focus Group Discussion
HDU: High Dependency Unit
ICU: Intensive Care Unit
KNRH: Kawempe National Referral Hospital
LBW: Low Birth Weight
MFM: Maternal Fetal Medicine
NICU: Neonatal Intensive Care Unit
PNW: Postnatal Ward
SCD: Sickle Cell Disease
